# A Low Membrane Hsp70 Expression in Tumor Cells With Impaired Lactate Metabolism Mediates Radiosensitization by NVP-AUY922

**DOI:** 10.3389/fonc.2022.861266

**Published:** 2022-04-07

**Authors:** Melissa Schwab, Gabriele Multhoff

**Affiliations:** ^1^Radiation Immuno-Oncology Group, Center for Translational Cancer Research (TranslaTUM), School of Medicine, Klinikum Rechts der Isar, Technical University of Munich (TUM), Munich, Germany; ^2^Department of Radiation Oncology, School of Medicine, Klinikum Rechts der Isar, Technical University of Munich (TUM), Munich, Germany

**Keywords:** *LDHA/B* double knockout, stress response, membrane Hsp70, radiosensitization, Hsp90 inhibitor NVP-AUY922

## Abstract

As overexpression and membrane localization of stress proteins together with high lactate levels promote radioresistance in tumor cells, we studied the effect of the Hsp90 inhibitor NVP-AUY922 on the cytosolic and membrane expression of heat shock proteins (HSPs) and radiosensitivity in murine melanoma (B16F10) and human colorectal (LS174T) wildtype (WT) and *lactate dehydrogenases A/B* double knockout (LDH^−/−^) tumor cells. Double knockout for *LDHA/B* has been found to reduce cytosolic as well as membrane HSP levels, whereas treatment with NVP-AUY922 stimulates the synthesis of Hsp27 and Hsp70, but does not affect membrane Hsp70 expression. Despite NVP-AUY922-inducing elevated levels of cytosolic HSP, radiosensitivity was significantly increased in WT cells and even more pronounced in LDH^−/−^ cells. An impaired lipid metabolism in LDH^−/−^ cells reduces the Hsp70 membrane-anchoring sphingolipid globotriaosylceramide (Gb3) and thereby results in a decreased Hsp70 cell surface density on tumor cells. Our results demonstrate that the membrane Hsp70 density, but not cytosolic HSP levels determines the radiosensitizing effect of the Hsp90 inhibitor NVP-AUY922 in LDH^−/−^ cells.

## Introduction

Many tumor cell types including colorectal carcinoma and melanoma, exhibit an increased synthesis of heat shock proteins (HSPs) such as Hsp90, Hsp70 and Hsp27 which in turn promotes tumor progression, malignant transformation and therapy resistance (1). In recent years, the therapeutic potential of several different HSP-targeting drugs has been tested in preclinical and clinical trials (2). Although, the Hsp90 inhibitor AUY-NVP922 exhibited promising radiosensitizing potential by impairing the DNA damage repair and the cell cycle, not only in different tumor cell entities including lung cancer cells, uterine cervical carcinoma, head and neck squamous cell carcinoma and colorectal carcinoma cells but also in a human head and neck squamous cell carcinoma xenograft model (3–5), its efficacy is limited due to its hepatotoxicity and a compensatory upregulation of the transcription of other HSPs, especially the major stress-inducible, anti-apoptotic Hsp70. As a consequence, combined treatment strategies with inhibitors targeting different HSP families concomitantly are currently under investigation, although clinical data are not yet available (2).

Our laboratory has previously demonstrated that a pharmacological inhibition of the lactate dehydrogenase (LDH) as well as a *lactate dehydrogenase A/B* (LDHA/B) double knockout (LDH^−/−^) has the capacity to decrease the expression of Hsp90, Hsp70 and Hsp27 and thereby can increase the radiosensitivity in cancer cells (6). An increased LDH activity causes high lactate concentrations and an acidic tumor microenvironment which further enhances tumor growth (7), suppresses immune cell functions including effector T and NK cells (8–10), correlates with an aggressive tumor phenotype and increases the risk of metastatic spread and tumor recurrence (11).

Compared to normal cells, tumor cells frequently overexpress Hsp70 in the cytosol and present it on their plasma membrane in a tumor-specific manner. A high cell surface density of Hsp70 stabilizes plasma membranes of tumor cells and thereby contributes to cell survival and radioresistance (12–14). Herein, we assessed the mechanism(s) *via* which an impaired lactate metabolism in combination with an Hsp90 inhibition impacts the stress protein expression and membrane localization of tumor cells in context with their radiosensitivity.

## Materials and Methods

### Cells and Cell Culture

The wildtype (WT) B16F10 murine melanoma (ATCC^®^ CRL-6475TM; ATCC, Manassas, VA, USA) and LS174T human colorectal adenocarcinoma (ATCC^®^ CL-188™; ATCC, Manassas, VA, USA) cell lines and their *LDHA/B* double knockout (LDH^−/−^) counterparts (kindly provided by Marina Kreutz and Jacques Pouyssegur (15) were grown in complete growth medium, consisting of Rosewell Park Memorial Institute (RPMI)-1640 medium (Sigma-Aldrich, St. Louis, MO, USA) or high glucose Dulbecco`s Eagle`s Minimum Essential Medium (DMEM) (Sigma-Aldrich) respectively, supplemented with 10% v/v heat inactivated fetal bovine serum (FBS, Sigma-Aldrich), 1% antibiotics (10,000 IU/mL penicillin, 10 mg/mL streptomycin, Sigma-Aldrich), 2 mM L-glutamine (Sigma-Aldrich) and 1 mM sodium pyruvate (Sigma-Aldrich). Cells were routinely checked and confirmed negative for mycoplasma contamination.

### Reagents and Treatment

A stock solution (10 mM) of the Hsp90 inhibitor NVP-AUY922 (Santa Cruz Biotechnology, Dallas, TX, USA) was prepared in dimethyl sulfoxide (DMSO) and further diluted in phosphate buffered saline (PBS). Control cells were incubated with the respective amounts of DMSO. Cells were treated with NVP-AUY922 for 24 h.

### Western Blot Analysis

Cells were lysed in Radioimmunoprecipitation Assay (RIPA) buffer containing 50 mM Tris-HCl (pH 8.0), 150 mM NaCl, 1 mM EDTA, 1% v/v Triton-X-100, 0.1% w/v sodium dodecyl sulphate (SDS), 0.5% w/v sodium deoxycholate, protease inhibitor cocktail (Roche, Basel, Switzerland). The protein amount was measured using the Pierce™ BCA Protein Assay Kit (Thermo Fisher Scientific, Waltham, MA, USA). Proteins were separated by SDS-PAGE, transferred on nitrocellulose membranes and detected by immunoblotting with the following primary and secondary antibodies: Hsp27 (NBP2-32972, clone G3.1, Novus Biologicals, Centennial, CO, USA), Hsp70 (clone cmHsp70.1, murine IgG1, multimmune GmbH, Munich, Germany), LDHA (NBP1-48336, rabbit polyclonal, Novus Biologicals), LDHB (NBP2-53421, rabbit polyclonal, Novus Biologicals), AKT (9272S, rabbit, Cell Signaling Technology, Danvers, MA, USA), ß-Actin (A2228, clone AC-74, Sigma-Aldrich), horseradish peroxidase (HRP)-conjugated rabbit anti-mouse immunoglobulins (P0260, Dako-Agilent, Santa Clara, CA, USA) and HRP-conjugated swine anti-rabbit immunoglobulins (P0217, Dako-Agilent). The Pierce™ ECL Western Kit (Thermo Fisher Scientific) was used to detect immune complexes which were then imaged digitally (ChemiDoc™ Touch Imaging System, Bio-Rad, Hercules, CA, USA). The Fiji software (16) was used for quantifying Western Blot signals.

### Lactate Dehydrogenase (LDH) Activity Measurement

LDH activity was measured using the Lactate Dehydrogenase Activity kit (Sigma-Aldrich) according to the manufacturer’s protocol.

### Cell Counting

Cell count and viability were determined using a Sigma-Aldrich Cell Counting Kit-8 (CCK-8), following the manufacturer’s protocol.

### Irradiation

Tumor cells were irradiated with a single dose of 0 Gy (sham), 0.5 Gy, 1 Gy and 2 Gy using the Gulmay RS225A device (Gulmay Medical Ltd., Camberley, UK) at a dose rate of 1.1 Gy/min (15 mA, 200 kV).

### Clonogenic Assay

Tumor cells were seeded into 12-well plates and one day later they were treated with 5 nM NVP-AUY922 for 24 h and then irradiated with the indicated doses. After irradiation cells were cultured in fresh, drug-free medium. After 9-10 days colonies were washed with PBS, fixed with ice-cold methanol and stained with 0.1% w/v crystal violet. The number of colonies consisting of ≥ 50 cells were counted automatically by a Bioreader^®^ 3000 (Bio-Sys GmbH, Karben, Deutschland). Survival curves were fitted to the linear quadratic model using SigmaPlot (Systat Software Inc, San Jose, CA, USA).

### Analysis of Membrane Hsp70 (mHsp70) Expression by Flow Cytometry

The membrane Hsp70 (mHsp70) phenotype was analyzed by flow cytometry using the FITC-conjugated cmHsp70.1 monoclonal antibody (mAb, IgG1, multimmune GmbH, Munich, Germany) on a FACSCalibur flow cytometer (BD Biosciences, Heidelberg, Germany). Tumor cells (0.2 x 10^6^ cells) were washed with flow cytometry buffer (PBS/10% v/v fetal bovine serum, FBS) and incubated either with the cmHsp70.1 mAb or with an isotype matched FITC-labeled control immunoglobulin (mouse IgG1 FITC, 345815, BD Biosciences) on ice in the dark for 30 min. After a second washing step, viable cells (propidium iodide negative cells) were gated upon, and the proportion of positively stained cells were analyzed.

### Statistics

Each experiment was performed independently at least 3 times (biological replicates). Comparative analysis of two or multiple groups was carried out using the Student’s t-test or the Tukey Test respectively (*p ≤ 0.05, **p ≤ 0.01, ***p ≤ 0.001). Data are presented as mean values with standard deviation (SD).

## Results

### Hsp90 Inhibition by NVP-AUY922 Increases Cytosolic Hsp70 and Hsp27 Expression in B16F10 and LS174T WT and LDH^−/−^ Cells

**Figure 1:**
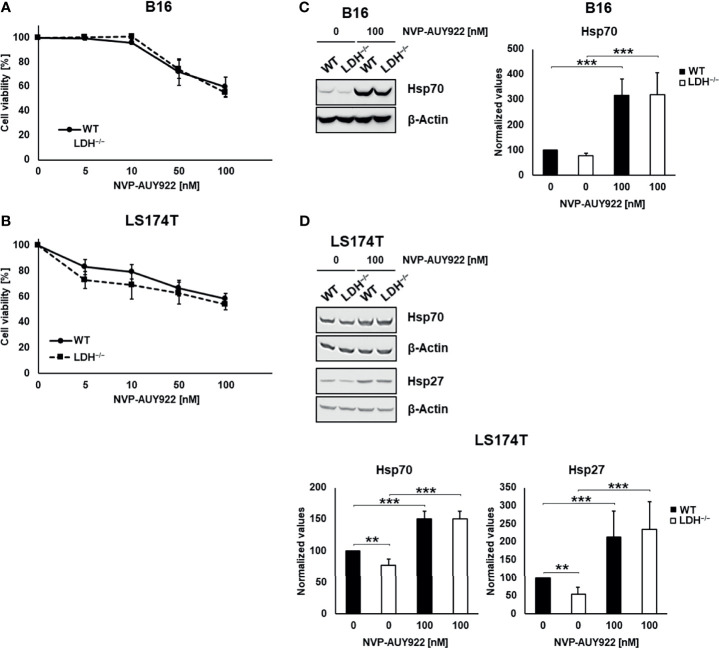
Hsp90 inhibition reduces cell viability in a concentration-dependent manner and increases cytosolic Hsp70 and Hsp27 levels. **(A, B)** Toxicity assay of B16F10 **(A)** and LS174T **(B)** WT and LDH^−/−^ cells treated with NVP-AUY922 (0, 5, 10, 50, 100 nM) for 24 h. **(C, D)** Representative immunoblot showing intracellular Hsp70, Hsp27 and β-Actin levels in B16F10 **(C)** and LS174T **(D)** cells upon treatment with NVP-AUY922 (100 nM) for 24 h. Quantification of the heat shock protein (HSP) levels are shown in the adjacent bar chart (**p ≤ 0.01, ***p ≤ 0.001).

The radiosensitizing effects of the Hsp90 inhibitor NVP-AUY922 was studied using murine (B16F10) and human (LS174T) wildtype (WT) and CRISPR/Cas9 *lactate dehydrogenases A/B* (LDH^−/−^) double knockout tumor cells with an impaired lactate metabolism (6, 15). The Hsp90 inhibitor NVP-AUY922 induced a comparable and concentration-dependent reduction in the viability of WT and LDH^−/−^ tumor cells (B16F10, LS174T; **Figures 1A, B**). In line with our previous data, a *LDHA/B* double knockout significantly reduced the cytosolic Hsp70 and Hsp27 expression (**Figures 1C, D**) (6). Despite significant differences in their basal levels of HSP expression, NVP-AUY922 caused a comparable upregulation of intracellular Hsp70 and Hsp27 in WT and LDH^−/−^ cells above the initial levels of WT cells (**Figures 1C, D**). Due to a very low Hsp27 expression, Hsp27 levels could not be quantified in B16F10 cells.

### Low Hsp90 Inhibitor Concentration Potentiates Radiosensitivity Especially in LDH^−/−^ Cells

**Figure 2:**
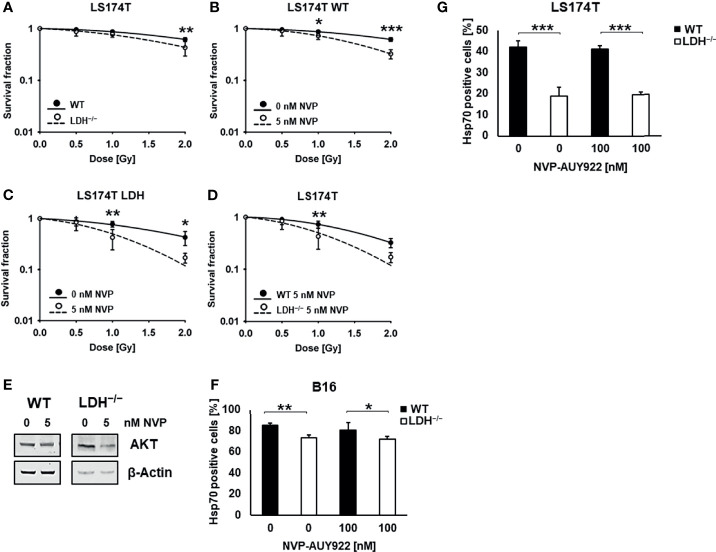
Hsp90 inhibition potentiates radiosensitivity in LS174T cells. **(A)** Colony forming assay of LS174T WT and LDH^−/−^ cells after irradiation with 0, 0.5, 1 and 2 Gy (**p ≤ 0.01). Colony forming assay of LS174T WT **(B)** and LDH^−/−^ **(C)** cells after treatment with a low concentration of NVP-AUY922 (5 nM) for 24 h and irradiation with 0, 0.5, 1 and 2 Gy (*p ≤ 0.05, **p ≤ 0.01, ***p ≤ 0.001). **(D)** Comparison of WT and LDH^−/−^ cells treated with a low dose of NVP-AUY922 (5 nM) (**p ≤ 0.01). **(E)** Representative immunoblot showing the expression of AKT and β-Actin in LS174T cells upon treatment with NVP-AUY922 (5 nM) for 24 h. **(F, G)** Membrane Hsp70 expression on B16F10 **(F)** and LS174T **(G)** cells treated with 100 nM NVP-AUY922 for 24 h, as determined by flow cytometry using the cmHsp70.1 mAb. The proportion of positively stained cells is shown (*p ≤ 0.05, **p ≤ 0.01, ***p ≤ 0.001).

In line with previous data, clonogenic cell survival assays revealed that LS174T WT cells are significantly more radioresistant than LDH^−/−^ cells (**Figure 2A**) (6). Despite elevated cytosolic HSP levels a low concentration of the Hsp90 inhibitor NVP-AUY922 (5 nM) increased radiosensitivity in WT and LDH^−/−^ cells. This radiosensitizing effect was significantly more pronounced in LDH^−/−^ cells (**Figures 2B–D**). Since a low concentration of 5 nM NVP-AUY922 completely inhibited clonogenic cell survival of B16F10 cells (**Supplementary Figure 1**) an additive radiosensitizing effect could not be shown in this cell line.

As demonstrated in **Table 1**, the D_50_ value of NVP-AUY922-treated WT versus LDH^−/−^ cells was 1.54 Gy and 1.0 Gy, respectively, and the sensitizing enhancement ratio (SER) was greater 1.20 (1.58 and 1.79, respectively) in both cell types. A downregulation of the Hsp90 client protein AKT (**Figure 2E**) confirmed the activity of the Hsp90 inhibitor NVP-AUY922 at a low concentration of 5 nM.

**Table 1:** Summary of radiobiological parameters depicted in **Figures 2A–D**.

LS174T	D_50_ [Gy]^a^	SER^b^	α [Gy^-1^]^c^	β [Gy^-1^]c
**WT**				
0 nM NVP	2.43	1.00	0.04	0.10
5 nM NVP	1.54	**1.58**	0.08	0.24
**LDH^−/−^**				
0 nM NVP	1.79	1.00	0.14	0.14
5 nM NVP	1.00	**1.79**	0.32	0.37

a D_50_, dose [Gy] required for 50% inactivation of a tumor cell population.

b SER, Sensitizing enhancement ratio = D_50_ (control)/D_50_ (drug treatment). A SER greater than 1.20 indicates a radio sensitization (indicated in bold).

c α and β values were derived from the linear quadratic equation f = exp(-α*x-β*x^2^).

In contrast to the cytosolic Hsp70 levels, the low membrane Hsp70 expression (6) remained unaltered low by Hsp90 inhibition in LDH^−/−^ B16F10 and LS174T tumor cells (**Figures 2F, G**).

## Discussion

Since many cells of different tumor entities including lung, breast, pancreatic and colorectal overexpress HSPs and are thus more resistant to therapy including radiotherapy, efforts are being made to develop HSP inhibitors (1, 17, 18). Although the synthetic, isoxazole/resorcinol-based Hsp90 inhibitor NVP-AUY922 has shown promising results in tumor cell lines and a human head and neck squamous cell carcinoma xenograft model (3, 5), its hepatotoxicity and a compensatory upregulated expression of anti-apoptotic HSPs limits its broader clinical application. The effectiveness of combination therapies using inhibitors targeting different HSP have therefore recently been investigated, although clinical data are still missing (2, 19). Our previous data demonstrated that combining the Heat Shock Factor 1 (HSF1) knockdown with Hsp90 inhibition using NVP-AUY922 radiosensitizes H1339 human lung cancer cells by impairing the DNA double-strand break repair (20). Furthermore, we have shown recently that targeting the lactate/pyruvate metabolism in cancer cells by a pharmacological or genetic inhibition of *LDHA/B* results in decreased cytosolic Hsp90, Hsp70 and Hsp27 levels and a reduced membrane Hsp70 expression (6). Therefore, we studied the radiosensitization effect of NVP-AUY922 in B16F10 and LS174T cells having an impaired lactate metabolism, and correlated radiosensitization with the cytosolic expression of different HSPs including Hsp27 and Hsp70 and the membrane Hsp70 positivity. A *LDHA/B* double knockout diminishes the HSP transcription and thereby reduces the cytosolic amounts of HSF1-regulated Hsp27 and Hsp70 (**Figures 1C, D**). However, the addition of NVP-AUY922 reversed this beneficial effect and resulted in a significant upregulation of cytosolic Hsp70 and Hsp27 in both, WT and LDH^−/−^ cells, even highly above initial levels (**Figures 1C, D**).

In contrast to the elevated cytosolic HSP levels, membrane Hsp70 expression remained unaffected by Hsp90 inhibition in both tumor cell types (**Figures 2F, G**). Tumor cells with an impaired lactate metabolism had a significantly lower membrane Hsp70 expression than WT cells after Hsp90 inhibition. Since the radiosensitizing effect of NVP-AUY922, even at low concentrations, was significantly more pronounced in LDH^−/−^ compared to WT cells (**Figures 2B–D**), despite the fact that both cell types exhibited comparably high cytosolic HSP levels, we propose that the increased radiosensitivity of LDH^−/−^ cells is associated with a reduced membrane Hsp70 positivity (**Figures 2F, G**) (6). The localization and anchorage of Hsp70 on the plasma membrane of tumor cells is enabled by a spontaneous interaction of Hsp70 with negatively charged sphingolipids including sulfogalactosyl ceramide (21) or globotriaosylceramide Gb3 (22) which are elevated in tumor cells and reside in cholesterol rich domains also termed lipid rafts (23). Atomic force microscopy studies (24) as well as the formation of ion conductance channels (25, 26) revealed a dimerization/clustering of Hsp70 in artificial lipid membranes which may affect the stability/fluidity of lipid membranes (27–29). Interference with the lactate/pyruvate metabolism results in an altered lipid metabolism (6) which also affects the production of Gb3. A reduction in the amount of the Hsp70-anchoring glycolipid Gb3 causes a significant decrease in the amount of plasma membrane-bound Hsp70 in LDH^−/−^ cells compared to WT cells. It remains to be determined whether an interference of the lactate/pyruvate metabolism also affects the trafficking of cytosolic Hsp70 to the plasma membrane and the release of Hsp70 in exosomes (30) into the extracellular milieu. Transport inhibitor studies revealed that membrane transport and exosomal export of Hsp70 are mediated *via* a non-classical liposomal but not a classical ER/Golgi pathway (31). Live cell STED nanoscopy has demonstrated that tumor cell-to-tumor cell connections are enabled by tunneling nanotubes that originate form membrane Hsp70 residing in cholesterol rich microdomains (32). It is conceivable that these nanotubes and cell interactions might also be impaired by an interference with the lactate metabolism.

A plasma membrane expression of Hsp70 on tumor cells correlates with the localization of Hsp70 in lysosomal membranes (33). Functionally, Hsp70 not only stabilizes plasma but also lysosomal membranes and thereby mediates resistance to chemical and/or physical-induced membrane permeabilization, such as anticancer drugs or radiation (13, 33, 34). Murakami et al. have demonstrated that not only cytosolic, but also plasma membrane-bound Hsp70 affects radiosensitivity (14). In this study, we demonstrate that the membrane Hsp70 status, not cytosolic Hsp70 levels, regulated by the lactate/pyruvate metabolism, determines the radiosensitizing effect of the Hsp90 inhibitor NVP-AUY922 in tumor cells.

Based on these findings, combining LDH and Hsp90 inhibition might provide a promising strategy to combat radioresistance, however further studies are necessary to identify more potent LDH inhibitors for clinical use with an improved efficacy, higher stability and lower off-target effects (35). The clinically approved, nonsteroidal anti-inflammatory drug (NSAID) diclofenac could be a potential candidate for efficiently inhibiting LDH activity (35–38).

## Data Availability Statement

The original contributions presented in the study are included in the article/**Supplementary Material**. Further inquiries can be directed to the corresponding author.

## Author Contributions

Conceptualization, MS and GM. Methodology, MS. Investigation, MS. Writing—original draft preparation, MS. Writing—review and editing, GM. Supervision, GM. Project administration, GM. Funding acquisition, GM. All authors have read and agreed to the published version of the manuscript.

## Funding

This research was supported by grants of the DFG (KU3500/2-1, SFB824, STA1520/1-1) and by BMWi (ZF4320104AJ8, ZF4320102CS7).

## Conflict of Interest

The authors declare that the research was conducted in the absence of any commercial or financial relationships that could be construed as a potential conflict of interest.

## Publisher’s Note

All claims expressed in this article are solely those of the authors and do not necessarily represent those of their affiliated organizations, or those of the publisher, the editors and the reviewers. Any product that may be evaluated in this article, or claim that may be made by its manufacturer, is not guaranteed or endorsed by the publisher.
