# Supplementary material for: A Low Membrane Hsp70 Expression in Tumor Cells With Impaired Lactate Metabolism Mediates Radiosensitization by NVP-AUY922
Source: Front Oncol. 2022 Apr 7;12:861266. doi: 10.3389/fonc.2022.861266 (PMC9022188; doi:10.3389/fonc.2022.861266)
Supplement: Supplementary file 1 [file DataSheet_1.docx]

Supplementary Material


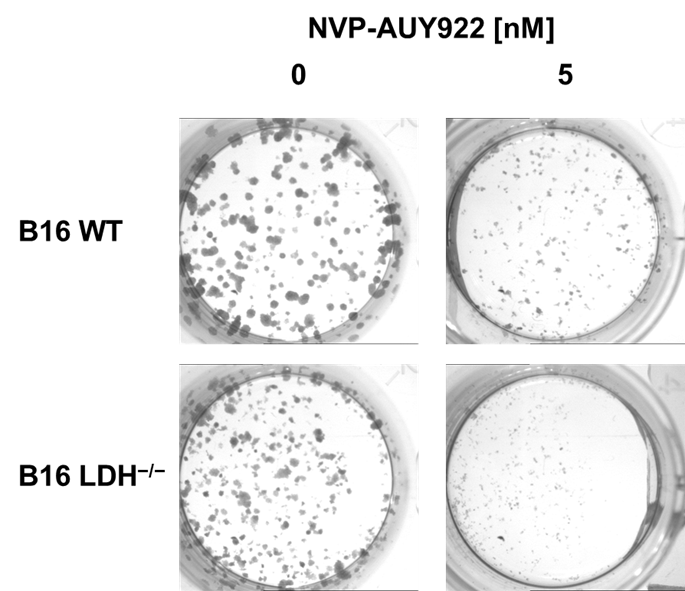


**Supplementary Figure 1.** Colony Forming Assay for B16F10 WT and LDH^−/−^ treated with NVP-AUY922. Representative images showing a colony forming assay of B16F10 WT and LDH^−/−^ treated with 5 nM NVP-AUY922 for 24 h.
